# Nexus Among Economic Growth, Education, Health, and Environment: Dynamic Analysis of World-Level Data

**DOI:** 10.3389/fpubh.2019.00307

**Published:** 2019-10-25

**Authors:** Suleman Sarwar, Majid Ibrahim Alsaggaf, Cao Tingqiu

**Affiliations:** ^1^School of Economics, Shandong University, Jinan, China; ^2^Finance and Economics Department, University of Jeddah, Jeddah, Saudi Arabia

**Keywords:** economic growth, education, health, carbon emission, panel data

## Abstract

The aim of current study is to examine the nexus among economic growth, education, health issues, and carbon emission for the panel of 161 countries. Education and health have confirmed insignificant coefficients for economic growth and carbon emission, which mention that higher education and better health conditions are not useful for boosting economic development and for controlling environmental degradation process. Empirical estimations have reported that higher capital investment leads to increase the economic process and carbon emission. Higher educational standard and capital investment helps to control the health issues, in the long- and short-run. On contrary, higher carbon emission creates health issues. The given results can provide support to the economic, social, and environmental policy makers during policy decisions. For example, the study suggests green financing and low carbon economy concept; the government and industries have to increase the investment on modern, energy efficient, and green technologies, which are useful for economic development, as well as to control the environmental degradation process.

## Introduction

This paper provides an insight into the supporting factors affecting economic growth, health issues, and carbon emission in a global perspective. Economic development is considered to be a high ranking issue in literature (1–5), as it is linked to various micro and macro issues, such as inflation, income, education, health, and environment, etc. However, a large number of empirical studies have investigated the influencing factors that lead to an increase in the economic growth. Solow (2) have reported that labor and capital are the key determinants of economic growth. Later, the Solow growth model has been extended by using education, health, carbon emission, energy consumption, industrialization, urbanization, taxes, foreign direct investment, etc. [e.g., (1, 6–16)], and these factors have confirmed mixed evidences due to the geographical differences, demographics, time periods, market structures, economic system, etc. (11, 14). Despite of large number of studies, there is no consensus about the most significant determinants of economic growth.

In addition to the previous studies, we extended the Solow growth model by augmenting education, health, and carbon emission. For this purpose, we examine the world level data, as well as grouped level analysis, to have more insight and indepth analysis. In sum, we investigate the impact of education, labor, and capital related factors on economic growth; labor related factors are health conditions, as it is difficult to manage large population, improve their life standard, and provide them with better health services. However, we assume that labor largely increases the health issues which reduces the labor productivity and economic development. Capital related factors can be carbon emission, as the developing countries have to strengthen their industrial infrastructure without much consideration given to environmental hazards. It seems that higher capital investment boosts economic growth, which leads to raise environmental degradation process, as confirmed by EKC hypothesis by a number of studies (17–20).

Firstly, while focusing on the education, there is a deep connection between education, labor and economic growth; higher education level produces the skillful labors, such skillful labors work efficiently and effectively which stimulates the economic growth, directly or indirectly. On other side, higher education leads to increase the economic growth through different channels: (i) higher education produces skilled labors that boost the economic growth, such economic growth attracts the local and foreign investors to invest their capital. (ii) Education helps the people in conjunction with developing enterprising skills, which helps in economic development. (iii) Advanced education produces creative and resourceful minds that explores ample opportunities to raise the capital for doing business (21, 22), which leads to expand the economic process. Hence, we can assume a significant relationship between education, labor, capital, and economic growth, either via a direct or indirect channel.

On the contrary, several researchers and economists have confirmed that early learning in school is insignificant for economic growth (23–25). There are multiple reasons for insignificance of education in economic growth: firstly, in underdeveloped and developing countries, the main purpose for education is to attain high salary jobs, instead of possessing high tech skills: secondly, in general, the countries follow traditional educational patterns that do not meet with need of time. Thirdly, most of the countries have a lack of resources, which does not guarantee economic development, however, education does not contribute to economic growth in such economies.

Secondly, economic growth is associated with labor productivity, which is linked to the health conditions of labors; healthy labor has a higher efficiency in performance, compared to the unhealthy labor (26–30). Healthy working individuals are able to live a pleasant life, which reduces the absence rate, improves the individual performance, and increases the economic output.

### Motivation and Contribution of Paper

The present study is motivated by a number of facts, which are inter-related; (i) the ongoing economic integration and financial crises affect the environment and health expenditures of the host country, (ii) similarly, fiscal budgets, industrial production, and economic developments are affected due to environmental hazards, health issues, and educational standards, (iii) it defines that the increase in health and environment related problems compels the governments to spend more money on improving human health, in the educational sector and in industrial treatment plans to protect the environment and health (31). In sum, economic growth, education, health, and environment are inter-related. Therefore, an empirical examination of growth, education, health, and environment, as a global perspective, and regional analysis are needed, which may provide us with innovative conclusions and policy reshaping.

The current study contributes in existing literature in multiple dimensions: firstly, the article extended the (2) growth model with education, health and carbon emission. Education is one of the most important factors for economic growth, it increases the economic growth through labor productivity and capital investment; labor productivity can be increased by replacing the uneducated labor with the educated labor, and such higher productivity leads to increase the economic growth of a country. In discussion of capital investment, education generates creative and productive minds that discover a number of opportunities to raise the capital for doing business, which expands the industrial infrastructure and enhances the economic process. On the contrary, it is also a matter of fact that the public get the education for the purpose of having a job, instead of generating creative minds and entrepreneurship. In such a scenario, the education level may be an insignificant factor for economic growth. However, we have to examine the impact of education on economic growth, whether significant or insignificant in global analysis. Secondly, as far as the relationship between education and health, education and carbon emission, we also investigate the importance of education in controlling the health issues and carbon emission, which has been missed by previous literature. Higher education is helpful to educate the public about human health and environmental health issues, and enable them to care for themselves, as well as to clean the environment. Thirdly, the paper investigates the role of economic growth toward education, health issues, and environment. To the best of authors' knowledge, the present study is the pioneer to investigate growth-health and environment nexus, in order to form the concerned innovative policies for different regions as income level and regional level, etc. Notably, the study investigated whether economic growth, health, and environment are inter-related and how different policies can be formed. Lastly, a significant contribution of the study is the empirical analysis for 161 countries as a global perspective and in-depth analysis for income, geographical, and OECD classification, in order to purpose fruitful implications for all studied countries.

## Intuition and Background Literature

The present study investigates three main relationships; (i) relationship between education in economic growth (ii) relationship between health conditions in economic growth (iii) relationship between carbon emission and economic growth.

Extensive empirical research is available that has investigated the role of educated work force to make variation in level of production that is resulted in economic development. Based on the theory of Schultz (32), the economic growth relies on strong educational standards that start from school level. An incredible change in economic growth has been found as there is an improvement in school education (32–35). The preschool and secondary school provides a base to produce a skilled and educated workforce (36). Additional schooling is highly recommended to improve the quality of labor as this action increases one fifth of GDP Abbas (37), and bidirectional causality has been found between education and economic growth. Besides, the strong educated system creates creatively minded individuals, who tend to prefer to own a business, instead of doing jobs. Such a business mind approach causes to increase the investment that strengthens the economy. Therefore, it has been concluded that there exists a significant relationship between human capital, investment, and economic growth (38).

However, it is also empirically tested that, along with high education standards, a high percentage of those educated in a population reduces the gap between employees, customers, and investors, etc. This removal of the communication gap dazzles investors toward a particular organization having a highly educated and skilled work force, so more opportunities are required to be furnished for a population to receive higher education. It has also been observed that, in urban areas, where universities are situated, more regional economic growth is found even at low magnitude (36, 39). In sum, there is a significant relationship among education, labor productivity, investment, and economic growth (30, 40).

Previous empirical investigations have documented a different relationship between health issues and labor productivity; it varies across regions, development phase, and income level, etc. In developing economies, it is evident that health status is positively correlated with economic growth but the same is ambiguous in industrialized economies (41). Furthermore, the economic growth is affected by health issues, particularly when the economy faces transitory changes. This investigation concludes that health issues are negatively associated to economy, as the health issues increases, economy growth deteriorates, and vice-versa. Rhum (42) reported that unfavorable health situation fully offset the growth of the economy. Based on some previous studies, the association between health conditions and macroeconomic factors, psychological health is considered to be a main determinant because mental condition leads to stress and affects workability of labor force. However, according to economic models, it is observed that the cost of medical care and disposable income influence investment in human capital, lifestyle, and stochastic shocks (43).

There is also an argument about budget and individual health; the standard of health conditions deteriorates during short term boost in economy (44). A similar result has been confirmed by Wang and Tapia Granados (29), which reported that economic development has a strong association with health condition. The absenteeism rate of healthier labors is quite low and such labor has confirmed higher productivity. However, the magnitude is different; there are different results that assess the direct or indirect relationship between health issues of labor and economic growth. It has been confirmed that labor productivity increases with good health (27, 45–47).

There exists a bidirectional relationship between health and economic growth; higher growth also has a significant impact on health. During higher growth periods, the employee demands fully efficient and effective workers that guarantee the higher productivity, which can cause stress, depression, and anxiety among workers. Such stress and depression affects the human health that resultantly decreases the labor productivity and slows down the economic process (48). Stevens et al. (49) has found that higher economic growth is possibly due to strong industrial infrastructure, whereas higher industrial production increases the production externalities. Such externalities have adverse impacts on human health, which leads to reduce the labor productivity. Later, Heutel and Ruhm (50), Davis et al. (51), and Luo et al. (52) have confirmed similar findings.

There are many factors, such as cultural background, social safety aggregate income, and active labor market policies acting as formal or informal mechanisms of insurance, which are systematically different across the county (53), and these factors may affect health conditions via economic growth. Whereas, the socioeconomic inequalities in mental health has widened (54–56). The changes in macroeconomic factors can lead to a change in the health conditions of a population, but a dissimilarity of magnitude for different age groups, ethnicity, education, and gender has been found (57, 58). Evidence has shown that during a recession the frequency of health issues raised in the workforce, particularly in the low salary class (59).

The relationship between economic growth and carbon emission has been discussed for a few decades. Kuznets (17) has presented Environmental Kuznets Curve (EKC) hypothesis. EKC mentions Inverted-U shape curve, mentioning that the higher growth in a developing country is highly responsible for carbon emission, as their main concern is to focus on growth, instead of environmental degradation. During the second stage, where the country reaches maximum growth level, its rate of exhaling emission reduces. In the third stage, the developed countries divert their attention toward environmentally friendly policies, as well as economic growth. However, in the third stage, the higher economic growth leads to reduce the carbon emission. Later, EKC hypothesis has been confirmed by a number of researchers (18, 19, 60, 61). In contrast, Ang (62), Long et al. (63), Abid (64), Zhang and Da (65), Esso and Keho (66), Mirza and Kanwal (67), Ahmad et al. (68), Bano et al. (69), and Le and Quah (70) have rejected the EKC hypothesis.

Empirical literature has confirmed that energy consumption is positively correlated with carbon emission. Some studies found unidirectional causality (71, 72). Whereas, some of the empirical studies have reported bidirectional causality between economic growth and carbon emission on cross sectional and longitudinal data (73). Since the industrial revolution, the economies of the world are using non-renewable resources to achieve maximum economic growth, but this serves to increase carbon emission and global warming. It has also been explored whether the developed countries are more likely to pursue energy consumption and have high rates of carbon emission because of more production (74–76). On the other side, some of the empirical evidences have reported a negative relationship between economic growth and carbon emission (8, 77–79).

Li et al. (80) reported that carbon emission increases air pollution and create health issues (respiratory and cardiovescular diseases) to the population, along with a hazard to environmental governance. However, the level of carbon emission increased with volume of production. Consequently, a raise in health issues leads to sickness, unemployment, poverty, and low living standards. The government has to infuse more funds to the health sector, as well as this situation, accompanied with lower per capita income (81, 82). To avoid health related problems, the government has to allocate more funds to the health sector. The allocation of huge funds to health sector reduces the focus on other sectors of economy, which ultimately leads to downtrend the economy. At the same time, the fast economic growth requires more energy to consume. With unbanization, increased population requires more sources of energy to consume the deteriorating environment. A survey during 2014–2016 shows a constanat increase in environmental pollution (80).

## Data and Materials

### Data and Models

The study investigates the nexus among economic growth, education, health, and carbon emission by augmenting Solow (2) growth model. Previously, a number of studies have reported the significance of Solow growth model, which confirmed the role of labor and capital for economic growth. After the recognition of Solow growth model (32–36), have documented the significance of education for economic growth, identifying that higher education standards have an important role in advancing economic development. Later, Feinstein (41), Faridi et al. (45), Umar (46), and Spiteri and Brockdorff (47) proposed the relevance of the health condition for labor productivity and economc growth. Afterwards, a number of studies have proclaimed the relationship between carbon emission and economic growth (76–78). In addition to the previous studies, we attempt to investigate the theoretical channel and nexus among studied variables; such as, does education effects the health, environmental degradation process, and economic growth process? Is health condition important for economic growth and for controlling the carbon emission? How economic development effects the health condition and environmental degradation process? To address these questions, we have emphasized on three models, as given:

(1) $$\begin{array}{l} Y=f\left(E,L,H,K,C\right) \end{array}$$

(2) $$\begin{array}{l} H=f\left(Y,E,L,K,C\right) \end{array}$$

(3) $$\begin{array}{l} C=f\left(Y,E,L,H,K\right) \end{array}$$

The models are constructed as:

(4) $$\begin{array}{l} Y_{it}=\beta_{0}+\beta_{1}Y_{it-1}+\beta_{2}E_{it}+\beta_{3}L_{it}+\beta_{4}H_{it}\\ \;+\beta_{5}K_{it}+\beta_{6}C_{it}+\varepsilon_{it} \end{array}$$

(5) $$\begin{array}{l} H_{it}=\beta_{0}+\beta_{1}H_{it-1}+\beta_{2}Y_{it}+\beta_{3}E_{it}+\beta_{4}L_{it}\\ \;+\beta_{5}K_{it}+\beta_{6}C_{it}+\varepsilon_{it} \end{array}$$

(6) $$\begin{array}{l} C_{it}=\beta_{0}+\beta_{1}C_{it-1}+\beta_{2}Y_{it}+\beta_{3}E_{it}+\beta_{4}L_{it}\\ \;+\;\beta_{5}H_{it-1}+\beta_{6}K_{it}+\varepsilon_{it} \end{array}$$

Where, *Y* represents the economic growth which is GDP (current US$), *E* is the education and is proxies with secondary education, *L* shows the labor, which is defined as total labor force. Health issue is represented as *H* and used the proxy incidence of tuberculosis (per 100,000 people); as tuberculosis is mainly caused by environmental changes (83, 84). K and C are capital and carbon emission, the definitions for these variables are Gross fixed capital formation (current LCU) and CO2 emissions (kt). For the econometric estimations, we have collected the data of 161 countries over the period of 2000–2016. The data of study variables have been collected from World Development Indicators (WDI).

We used the log transformations method to standardize the variables, which is widely used to handle the skewed data and puts it in an interpretable form. The log transformed models are mentioned below:

(7) $$\begin{array}{l} \ln\;Y_{it}=\beta_{0}+\beta_{1}\ln Y_{it-1}+\beta_{2}\ln E_{it}+\beta_{3}\ln L_{it}+\beta_{4}\ln H_{it}\\ \;+\;\beta_{5}\ln K_{it}+\beta_{6}\ln C_{it}+\varepsilon_{it} \end{array}$$

(8) $$\begin{array}{l} \ln\;H_{it}=\beta_{0}+\beta_{1}\ln H_{it-1}+\beta_{2}\ln Y_{it}+\beta_{3}\ln E_{it}+\beta_{4}\ln L_{it}\\ \;+\;\beta_{5}\ln K_{it}+\beta_{6}\ln C_{it}+\varepsilon_{it} \end{array}$$

(9) $$\begin{array}{l} \ln\;C_{it}=\beta_{0}+\beta_{1}\ln C_{it-1}+\beta_{2}\ln Y_{it}+\beta_{3}\ln E_{it}+\beta_{4}\ln L_{it}\\ \;+\;\beta_{5}\ln H_{it-1}+\beta_{6}\ln K_{it}+\varepsilon_{it} \end{array}$$

### Estimation Strategy

Firstly, we check the cross-sectional dependence of panel data; for this we have used Breusch and Pagan (85) LM test. For robustness, we also use Pesaran (86) cross sectional dependence test, having additional advantages over Breusch and Pagan (85) test; (i) it deals with pair-wise correlation coefficients instead of their squares; (ii) it provides robust results with single and multiple structural breaks in slop coefficients and in error variance; (iii) it accounts for the data having large *N* and short *T*.

Secondly, after examining the cross-sectional dependence, we have to find the stationarity in series of each variables for further estimations. In our case, we follow both, first generation and second-generation unit root test to confirm the stationarity in series. For first generation unit root test, we have utilized well known Augmented Dicky Fuller (ADF), Phillips–Perron (PP), and Maddala and Wu (87) estimation techniques. Pesaran (88) CIPS unit root test is considered for second generation unit root estimations.

In order to examine our primary hypothesis regarding the growth-health-environment nexus, we employed the system GMM technique and difference GMM techniques. The difference generalized method of moment (difference GMM), introduced by Arellano and Bond (89), considers the auto-regressive level (1 and 2) with individual-specific unobserved factors. The dynamic panel models, such as difference GMM, provide the consistent and reliable estimations (90). In the current study, the data consists of 161 countries (*N* = 161) and covers the period of 17 years (*T* = 17), in such case, where N is more than T, dynamic panel model is appropriate for estimations (91). Bond et al. (90) argued that GMM methods are able to correct unobserved country heterogeneity, omit variable bias and measurement error, and help to avoid endogeneity issues. Due to such advantages, we have employed the one step and two step system and difference GMM methods for full specification and regional analysis (income level, regions, and OECD levels). Such an approach could be beneficial in providing consistent and reliable regression results. The GMM techniques are employed as level equations, with robust standard errors. Further, to verify that our results are not spurious, we also employed the fixed effects and pooled OLS techniques with robust standard errors. The robust results of fixed effects and pooled OLS are reported along our primary estimations of GMM. Our primary models of estimation are presented in GMM equations as follows;

(10) $$\begin{array}{l} \ln\;Y_{it}=\alpha_{0}+\beta_{1}\ln Y_{it-1}+\delta \ln E_{it}+\gamma \ln H_{it}\\ \;+\sum_{j=1}^{3}\theta Z_{i,t}+\mu_{it}+\varepsilon_{it} \end{array}$$

(11) $$\begin{array}{l} \ln\;H_{it}=\alpha_{0}+\beta_{1}\ln H_{it-1}+\delta \ln Y_{it}+\gamma \ln E_{it}\\ \;+\sum_{j=1}^{3}\theta Z_{i,t}+\mu_{it}+\varepsilon_{it} \end{array}$$

(12) $$\begin{array}{l} \ln\;C_{it}=\alpha_{0}+\beta_{1}\ln C_{it-1}+\delta \ln Y_{it}+\gamma \ln E_{it}\\ \;+\sum_{j=1}^{3}\theta Z_{i,t}+\mu_{it}+\varepsilon_{it} \end{array}$$

Whereas, *Z*_*i,t*_ presents the vector of control variables taken in the model. μ_*i,t*_ shows the country specific effects and ε_*i,t*_ is the error term. While, ^γ^ captures the effect of primary independent variable toward the main dependent variables in our baseline specifications. The generalized method of moments (GMM) estimator provides the consistent parameter estimates for such econometric models, as it also captures the unobserved country-specific heterogeneity and endogeneity (89). Such an empirical methodology has been employed in a recent environment study of Saidi and Hammami (60) for their empirical work on 58 countries.

## Empirical Results and Discussion

### Cross-Sectional Dependence

Table 1 reports the findings of cross-sectional dependence among studied panels. The findings of Breusch-Pagan LM test, Pesaran scaled LM test, Bias-corrected scaled LM test, and Pesaran CD test have presented significant values, which confirms that null hypothesis of cross-sectional independence has been rejected in all statistics. Resultantly, we report the presence of cross-sectional dependence, which motivates us to reply on second generation unit root testing, instead of first-generation unit root estimations^1^.

**Table 1:** Cross sectional dependence results.

**Test**	**Y**	**E**	**L**	**H**	**K**	**C**
Breusch-Pagan LM	177473^***^	125304^***^	175394.8^***^	62636.01^***^	77425.3^***^	106600.2^***^
Pesaran scaled LM	1024.504^***^	699.4612^***^	1011.555^***^	309.0047^***^	729.521^***^	582.9261^***^
Bias-corrected scaled LM	1019.472^***^	694.4299^***^	1006.524^***^	303.9734^***^	644.759^***^	577.8949^***^
Pesaran CD	417.3057^***^	41.76783^***^	310.7172^***^	126.2689^***^	335.77^***^	119.5644^***^

***, **, * *Indicates the significance at 1%, 5%, and 10%, respectively*.

### Panel Unit Root Testing

Pesaran (88) CIPS second generation unit root test is used to examine the existence of stationarity in series. In Table 2, second generation unit root test has reported that all the variables are stationary; economic growth, education, firm's capital formation and carbon emission at 1% level, whereas, labor and health issues are significant at 10% level. As all the variables are stationary, this directs us to apply for long-run panel empirical estimation techniques (difference GMM and system GMM, Pooled OLS, fixed effect models, etc.).

**Table 2:** Panel Unit root testing.

**At Level**	**Y**	**E**	**L**	**H**	**K**	**C**
**Second generation unit root test**						
CIPS Pesaran (86)	−7.262^***^	−3.962^***^	−0.202^**^	27.428^**^	−2.359^***^	−5.57^***^
**First generation unit root tests**						
ADF test	646.755^***^	352.538^**^	403.053^***^	411.188^***^	426.298^***^	484.802^***^
PP test	1180.5^***^	1283.82^***^	973.981^***^	817.623^***^	543.99^***^	484.25^***^
Maddala and Wu (87)	637.94^***^	507.53^***^	1045.26^***^	611.92^***^	446.15^***^	319.99^**^

***, **, * *Indicates the significance at 1%, 5%, and 10%, respectively*.

### Full Specification Estimations

#### Economic Growth

In one-step difference GMM, we note significant and positive lagged economic growth, capital, and carbon emission, mentioning that higher firm capital formation and carbo emission tends to increase the economic growth. In case of a two-step difference GMM, previous economic growth and capital have confirmed significant and positive coefficients. Focusing on the coefficients of lagged economic growth, 0.7874 and 0.8032 are the coefficients of one-step difference GMM and two-step difference GMM, indicating that lagged economic growth has a consistent response in all empirical estimations.

Table 3 presents the results of Equation (7), where pooled OLS has confirmed the significant positive coefficients of lagged economic growth and carbon emission. The coefficient of lagged economic growth and carbon emission are 0.9533 and 0.0344, respectively, mentioning that higher lagged economic growth and carbon emission increases the economic growth. The positive relationship between carbon emission and economic growth is in line with previous studies (8, 70, 92–97). Afterwards, we employed fixed effect model to compare the estimations of pooled OLS and fixed effect model. In the case of fixed effect model, lagged economic growth, labor, capital and carbon emission all have significant positive coefficients, indicating that higher labor, capital, and carbon emission tends to increase the economic growth. The coefficients of lagged economic growth are 0.9533 and 0.7793 in pooled OLS and fixed effect model, respectively, which presents that the previous year's economic growth positively affects the current year economic development.

**Table 3:** Full specification estimations (Dependent variable: Economic Growth).

**Variables**	**Pooled** **OLS**	**FE**	**One-step diff-** **GMM**	**Two-step diff-** **GMM**	**One-step system-** **GMM**	**Two-step system-** **GMM**
Lag Y	0.9533^***^ (0.0043)	0.7793^***^ (0.0167)	0.7874^***^ (0.0166)	0.8032^***^ (0.0268)	0.8677^***^ (0.0107)	0.8677^***^ (0.0109
E	0.0083^*^ (0.0052)	−0.0081 (0.0195)	−0.0061 (0.0189)	−0.0137 (0.0315)	−0.0245 (0.0294)	−0.0247 (0.0306)
L	0.0005 (0.0051)	0.0668^**^ (0.0378)	0.0562 (0.0401)	0.0461 (0.0727)	0.0341 (0.0260)	0.0337 (0.0271)
H	−0.0124 (0.0099)	0.0271 (0.0247)	0.0257 (0.0248)	0.0291 (0.04300	0.0219 (0.0204)	0.0227 (0.0214)
K	0.0020^*^ (0.0013)	0.0824^***^ (0.0143)	0.0787^***^ (0.0129)	0.0806^***^ (0.0203)	0.0077^**^ (0.0030)	0.0078^**^ (0.0031)
C	0.0344^^***^^ (0.0043)	0.0467^**^ (0.0247)	0.0445^**^ (0.0242)	0.0392 (0.0381)	0.1074^***^ (0.0120)	0.1075^***^ (0.0123)
Constant	0.9985 (0.0988)	2.1516 (0.4758)			2.4998 (0.2294)	2.5026 (0.2362)
AR (2)			0.056	0.056	0.057	0.057
Sargan OIR			0.456	0.406	0.064	0.064

***, **, * *Indicates the significance at 1, 5, and 10%, respectively. Standard errors are reported in parenthesis. AR (2) shows the auto correlation in GMM models, while Sargan presents the validity of instruments*.

In doing the one-step system GMM and two-step system GMM, the coefficients of lagged economic growth appear to be 0.8677, which is in consistent with pooled OLS and fixed effect estimations. The findings of one-step and two-step system GMM are consistent, which report the significant and positive coefficients of capital and carbon emission, indicating that higher capital investment and carbon emission tend to increase the economic growth. The positive impact of capital and carbon emission on economic growth can be due to several factors, for example the higher carbon emission is caused by industrialization and urbanization, etc. In this scenario, the increasing trend of industrialization is due to higher capital investment, which on one side triggers the economic growth, and creates environmental issues. It means that higher capital investment is used for industrialization, which requires more energy and transportation means. However, this higher energy consumption through non-renewable sources and fuel consumption for transportation creates environmental issues, such as an increase in carbon emission.

#### Health

Table 4 mentions the impact of economic growth, education, labor, capital, and carbon emission on human health, as followed in Equation (8). Similarly to the analysis of Tables 3, 4 presents the findings of pooled OLS, fixed effect model, difference GMM, and system GMM, where the estimated results are more or less the same for all tests. The coefficients of lagged dependent (health issues) variable has confirmed the significant and positive response in all empirical techniques for full specification estimations, inferring that the current period health problems incite to increase future health issues.

**Table 4:** Full specification estimations (Dependent variable: Health Issues).

**Variables**	**Pooled** **OLS**	**FE**	**One-step diff-** **GMM**	**Two-step diff-** **GMM**	**One-step system-** **GMM**	**Two-step system-** **GMM**
Lag H	0.8918^***^ (0.0171)	0.7035^***^ (0.0277)	0.4203^**^ (0.1720)	0.4607^**^ (0.2018)	0.8753^***^ (0.0232)	0.8754^***^ (0.0232)
Y	−0.0012 (0.0024)	−0.0014 (0.0101)	0.0142 (0.0189)	0.0151 (0.0133)	−0.0021 (0.0059)	−0.0021 (0.0058)
E	−0.0054^**^ (0.0026)	−0.0338^***^ (0.0092)	0.0574 (0.0362)	0.0535 (0.0413)	−0.0063^**^ (0.0027)	−0.0062^**^ (0.0027)
L	0.0018 (0.0026)	0.0148 (0.0186)	−0.0178 (0.0480)	−0.0351 (0.0354)	0.0009 (0.0035)	0.0008 (0.0036)
K	−0.0019^**^ (0.0009)	0.0057 (0.0071)	0.0087 (0.0124)	0.0077 (0.0144)	−0.0020^**^ (0.0011)	−0.0020^**^ (0.0011)
C	0.0066^**^ (0.0028)	−0.0153 (0.0104)	0.0206 (0.0637)	0.0325 (0.0622)	0.0090^*^ (0.0050)	0.0090^**^ (0.0049)
Constant	0.5727 (0.1011)	0.5382 (0.2409)			0.6881 (0.1387)	0.6879 (0.1371)
AR (2)			0.225	0.209	0.140	0.140
Sargan OIR			0.320	0.320	0.555	0.555

***, **, * *Indicates the significance at 1, 5, and 10%, respectively. Standard errors are reported in parenthesis. AR (2) shows the auto correlation in GMM models, while Sargan presents the validity of instruments*.

One-step and two-stem System GMM have reported the significant and negative coefficients of education, proposing that higher education level tends to decrease the health issues. This finding is similar with Zajacova and Lawrence (98). The coefficients of capital are significant and negative, at 10% level, mentioning that higher capital investment leads to decrease the health issues. This result indicates that higher capital investments are not responsible for health damages but help to reduce the health issues. Focusing on the carbon emission and health relationship, it is found that higher carbon emission is a significant contributor to health problems. Such a positive result is in line with the previous literature [such as, (31, 99, 100)].

#### Carbon Emission

For Equation (9), in undertaking carbon emission as dependent variables, we have noticed that economic growth is significant and positive in fixed effect estimation, while education is significant and positive in pooled OLS, fixed effect, and difference GMM estimations, as reported in Table 5. After reviewing the coefficients of lagged carbon emission, we may conclude that the previous periods carbon incites to increase the future carbon. However, the significant response is consistent in all empirical estimations with different magnitudes.

**Table 5:** Full specification estimations (Dependent variable: carbon emission).

**Variables**	**Pooled** **OLS**	**FE**	**One-step diff-** **GMM**	**Two-step diff-** **GMM**	**One-step system-** **GMM**	**Two-step system-** **GMM**
Lag C	0.9871^***^ (0.0034)	0.7767^***^ (0.0205)	0.8333^***^ (0.0545)	0.8311^***^ (0.0586)	0.9997^***^ (0.0079)	0.9996 (0.0074)
Y	0.0013 (0.0033)	0.0254^**^ (0.0128)	0.0174 (0.0134)	0.0161 (0.0139)	−0.0085 (0.0084)	−0.0086 (0.0079)
E	0.0095^***^ (0.0032)	0.0992^***^ (0.0166)	0.0810^***^ (0.0260)	0.0789^***^ (0.0274)	0.0054 (0.0046)	0.0056 (0.0049)
L	0.0004 (0.0028)	0.0412 (0.0320)	0.0197 (0.0306)	0.0199 (0.0319)	0.0008 (0.0032)	0.0007 (0.0033)
H	−0.0152^*^ (0.0095)	−0.0013 (0.0215)	−0.0034 (0.0204)	−0.0002 (0.0200)	−0.0220 (0.0152)	−0.0212 (0.0138)
K	0.0020^**^ (0.0010)	0.0232^**^ (0.0092)	0.0198^**^ (0.0089)	0.0200^**^ (0.0087)	0.0024^**^ (0.0009)	0.0024 (0.0009)
Constant	−0.0859 (0.0813)	−2.5131 (0.3912)			0.1867 (0.1784)	0.1836 (0.1680)
AR (2)			0.381	0.384	0.385	0.384
Sargan OIR			0.056	0.056	0.142	0.142

***, **, * *Indicates the significance at 1, 5, and 10%, respectively. Standard errors are reported in parenthesis. AR (2) shows the auto correlation in GMM models, while Sargan presents the validity of instruments*.

Surprisingly, all the variables are insignificant, except capital which is significant and positive, reporting that higher business activities are responsible for environmental degradation. This result is in line with previous studies (70, 95, 101–103).

Table 6 reports the empirical results for full specification by using system GMM, where the first to third column use economic growth, health issues and carbon emission as dependent variables, respectively. There is a significant and positive relationship between capital investment and economic growth, which is inconsistent with our main empirical results. This finding suggests that a significant increase in capital investment boosts the industrial production, which further upsurges the economic growth. However, the governments have to provide easy grounds to the investors to enhance the business opportunities.

**Table 6:** Full specification estimations (system GMM).

	**Y**	**H**	**C**
Y	–	−0.0172	−19.2538
E	−0.1869	−0.0494^**^	12.4436
L	0.2550	0.0067	1.6378
H	0.1713	–	−47.4913
K	0.0586^***^	−0.0161^**^	5.2636
C	0.8125^***^	0.0719^*^	–
AR (2)	0.054	0.074	0.064
Sargan OIR	0.456	0.416	0.016

***, **, * *Indicates the significance at 1, 5, and 10%, respectively. AR (2) shows the auto correlation in GMM models, while Sargan presents the validity of instruments*.

In account of health issues as a dependent variable, it is confirmed that education and capital have a significant and negative association with health issues, mentioning that a higher level of education and firm investments scale down the health issues. In the case of carbon emission as a dependent variable, system GMM analysis has reported that all the variables are insignificant, indicating that economic growth, education, labor, health and firm investment have no impact on carbon emission. We observe that the insignificant relationship between economic growth and carbon emission is similar to some recent studies (104, 105), while it contradicts some other studies [such as (64, 68, 106, 107)]. Accounting for this contradiction, we have to further analyze the association among study variables through using geographical based analysis and countries income-based analysis, etc., which reinforce our empirical analysis.

### Sub-group Analysis

After conducting the full specification analysis, we further segregated the data into regions, income level and OECD based countries to reinvestigate the previous findings. The sub-group analysis is conducted by employing the system GMM technique.

#### Economic Growth

Table 7 reports the effect of studied variables on economic growth, where the coefficients of lagged economic growth have reported significant and positive values in all regional levels, income levels, and OECD based analysis, except for North America and South Asia, mentioning that higher economic growth leads toward economic prosperity. Lower-middle income countries report the highest coefficient with the value of 0.9466, which represents that lower-middle income countries have a greater potential to grow in future with lagged economic growth (15).

**Table 7:** Regional and group wise analysis—Two-step system-GMM (Dependent variable—Economic Growth).

**Groups**	**Lag Y**	**E**	**L**	**H**	**K**	**C**	**Constant**
**Region**							
East Asia and Pacific	0.9214^***^	0.0212	−0.0046	0.0320	0.0011	0.0603	1.4382
Europe and Central Asia	0.9297^***^	0.0992	−0.1074	0.0944	−0.0046	0.0775^***^	1.5612^***^
Latin America and Caribbean	0.9212^***^	0.0273	−0.0031	−0.1302	0.0028	0.0243	1.3757
Middle East and North Africa	0.8994^***^	0.1533	−0.0301	0.0037	0.0227	0.0026	0.3966
North America	0.7968	0.0040	0.0180	0.0072	0.0377	1.2893	0.0014
South Asia	0.6067	−0.4342	−0.5354	1.2719	−0.2115	0.8684	22.8099
Sub-Saharan Africa	0.9240^***^	−0.0639	0.0137^*^	−0.0246	0.0062	0.0658^***^	1.4005^***^
**Income level**							
Low	0.9012^***^	0.0336	0.0414	−0.0117	0.0040	0.0160	1.1557
Lower-Middle	0.9466^***^	0.0258	0.0351	−0.0208	−0.0013	0.0084	1.0316^***^
Upper-Middle	0.8481^***^	0.0175	0.0288	0.0663	0.0125^*^	0.0920^**^	2.1987^***^
High	0.8955^***^	0.0789^**^	−0.0618^**^	0.0465	0.0100^***^	0.0710^***^	1.9096^***^
**OECD**							
OECD	0.9067^***^	0.1572	−0.1655	0.1426	0.0001	0.0956^***^	1.9049
nonOECD	0.8865^***^	0.0253	−0.0257	−0.1047	0.0123	0.0796^**^	2.8168

***, **, * *Indicates the significance at 1, 5, and 10%, respectively*.

As for the education, higher education in high income countries is one of the main factors of economic growth, which is insignificant in remaining sub-groups. The insignificance of education is contradicting with Valero and Van Reenen (36), Abbas (37), Sobiech (40), Breton (108), and Greer and Kuhlmann (30). However, the main reason for the significance of education in high income countries is the research based and advanced education structure that is designed according to the current needs. Further, the education quality in high income countries is based on research and the development of advanced technologies, instead of being focused on old and outdated technologies (109). On the contrary, low income countries follow traditional educational patterns and also face lack of resources, which does not guarantee economic development (110).

In our regional analysis, Sub-Saharan African countries have a significant and positive coefficient for labor, while high income countries have a significant and negative coefficient, and the remaining countries show an insignificant coefficient. In the case of Sub-Saharan African countries, positive coefficient mentions that higher labor force is one of the main causes of economic growth, which is being reported by previous researchers (15, 16). The reason for depending on labor force for economic growth is the lack of technology and dependence on a traditional way of production, which requires higher labors. In the case of a negative relationship for high income countries, the finding proposes that more labor in developed countries may become a burden on economy, as their economy is already utilizing its resources more efficiently. It seems to be a balance between labors employment, supply, and demand of goods, which helps to maintain the higher pace of economic growth, especially in high income countries. However, increasing the labor supply will affect the economy directly or indirectly. For example, an increase in labor supply may reduce the wages, which in turn lowers the consumption, and destabilizes the firm's production, profitability, industrial growth, and economic progress.

Health coefficients are insignificant, which suggests that health has no significant impact on economic growth. These findings are in line with Chansarn (111), who confirmed an insignificant relationship between human health and economic growth in G7 countries, western developed countries, eastern developed countries and eastern developing countries. Regarding the capital, upper-middle-, and high-income countries, which have significant and positive responses, suggesting that higher capital leads toward economic growth. In discussing the relationship between carbon emission and economic growth, significant, and positive coefficients are reported in Europe and Central Asia, Sub-Saharan African, upper-middle income, high income, OECD, and non-OECD countries. This mentions that higher carbon emission is due to some productive channels, such as industrialization, urbanization, and transportation, etc., which are the main sources for carbon emission, as well as the economic growth (112).

#### Health

In this section, we analyze the impact of lagged health issues, economic growth, education, labor, capital, and carbon emission on health issues, as reported in Table 8. Lagged health issues have significant and positive coefficients. Economic growth is significant and negative in Sub-Saharan African, lower-middle income, upper-middle income, and high-income countries, which proposes that higher economic development tends to reduce the health issues. This finding suggests that the countries having higher economic growth increase their health standards and provide better health facilities to the public, which reduce the health issues (47, 80, 113, 114). Another study by Bekun et al. (8) has discussed that higher economic growth helps to reduce the health issues.

**Table 8:** Regional and group wise analysis -Two-step system-GMM (Dependent variable—Health Issues).

**Groups**	**Lag H**	**Y**	**E**	**L**	**K**	**C**	**Constant**
**Region**							
East Asia and Pacific	0.8888^***^	0.0102	−0.0021	0.0039	−0.0026	−0.0068	0.2981
Europe and Central Asia	0.7880^***^	0.0083	−0.0034^**^	0.0027	0.0012	−0.0060	0.7293^***^
Latin America and Caribbean	0.9485^***^	0.0013	−0.0033^**^	0.0024	0.0001	−0.0007	0.2041
Middle East and North Africa	0.9356^***^	0.0006	−0.0020	−0.0007	−0.0013	0.0021	0.3319^**^
North America	−0.0298	0.1804	0.0221	0.0315	0.1105	0.0087	0.0062
South Asia	0.5311	0.0027	0.1229	−0.0093	0.2291	−0.3128	−5.1275
Sub-Saharan Africa	0.8969^***^	−0.0146^***^	−0.0056^*^	0.0045	0.0006	0.0087	0.7452^***^
**Income level**							
Low	0.8900^***^	0.0029	0.0080	0.0343	−0.0024	−0.0305	−0.1512
Lower-Middle	0.8531^***^	−0.0187^*^	−0.0027^*^	−0.0087	−0.0010	0.0221^**^	1.2103^***^
Upper-Middle	0.9333^***^	−0.0144^**^	−0.0054^**^	0.0075^*^	−0.0016	0.0042^*^	0.4870^**^
High	0.9491^***^	−0.0025^*^	0.0015	−0.0005	−0.0006	0.0027	0.2866^**^
**OECD**							
OECD	10.8666	0.0029	0.0167	−0.0092	0.0001	−0.0099	−44.1763
nonOECD	0.9311^***^	−0.0053	0.0006	0.0004	0.0035	0.0040	0.4100^**^

***, **, * *Indicates the significance at 1, 5, and 10%, respectively*.

Education has confirmed significant and negative coefficients in Europe and Central Asia, Latin America, and Caribbean, Sub-Saharan Africa, lower-middle income and upper-middle income countries. This finding shows that health issues can be covered by increasing the education, as educated persons are more concerned about health than uneducated persons (115, 116). Labor and capital are reporting insignificant coefficients, except upper-middle income where labor is significant and positive with weak magnitude. However, it can be concluded that labor and capital have no impact on health issues. For carbon emission, lower-middle income, and upper-middle income countries have significant and positive coefficients, with values of 0.0221 and 0.0042, which indicates that carbon emission is responsible for health issues in these regions. Farooq et al. (31) has confirmed a similar finding, in the case of China, which suggests a significant effect of carbon emission on health. Whereas, this increase in health issue leads to increase the aging population, which becomes a big issue for a country; aging population is considered as non-productive that is a burden on economy.

#### Carbon Emission

Table 9 presents the results by using carbon emission as a dependent variable, economic growth has reported significant and positive coefficients in East Asia and Pacific, Sub-Saharan Africa, upper-middle income, and OECD countries. This significant and positive relationship is consistent with previous studies [e.g., (61, 117, 118)], which have documented that, during growth process, the policy makers are much more concerned about continued growth instead of environmental protocols. However, during the development phase, these countries use cheap methods of energy generation, which less focused on industrial treatment plants, etc. For education and labor, the coefficients are insignificant in most of the sub samples, which are in line with our full sample estimations of system-GMM. The coefficients of health are insignificant, expect for Latin America and Caribbean. In estimations of capital, East Asia and Pacific, South Asia, and lower-middle income countries have significant and positive coefficients, indicating that higher capital is responsible for higher carbon emission, which confirms the full sample results.

**Table 9:** Regional and group wise analysis- Two-step system-GMM (Dependent variable—Carbon Emission).

**Groups**	**Lag C**	**Y**	**E**	**L**	**H**	**K**	**Constant**
**Region**							
East Asia and Pacific	0.9728^***^	0.0059^**^	0.0162	0.0064	−0.0187	0.0023^**^	−0.3206
Europe and Central Asia	0.9975^***^	−0.0053	0.0313^**^	−0.0250	0.0176	−0.0009	0.0572
Latin America and Caribbean	0.8325^***^	0.0224	0.0732	0.0809^*^	0.5919^*^	−0.0080	−4.6501^*^
Middle East and North Africa	1.0043^***^	0.0099	0.0144	−0.0417	−0.0489	0.0025	0.3474
North America	0.0000	−0.1481	0.0000	0.2144	0.0000	0.1707	0.0000
South Asia	0.3386	−1.1149	0.7887	0.0443	−0.0546	0.8159^**^	−5.1750
Sub-Saharan Africa	0.9490^***^	0.0278^**^	0.0173	0.0015	0.0025	0.0005	−0.8112
**Income level**							
Low	0.9830^***^	0.0218	0.0077	−0.0052	−0.0137	0.0020	−0.4454
Lower-Middle	0.9564^***^	−0.0018	0.0189	0.0277	0.0243	0.0026^**^	−0.6755
Upper-Middle	0.9426^***^	0.0216^**^	0.0244^*^	0.0218	−0.0715	−0.0028	−0.5917
High	1.0155^***^	−0.0150	0.0070	−0.0133	−0.0058	0.0025	0.4008^*^
**OECD**							
OECD	0.9916^***^	0.0185^**^	0.0180	0.0046	−0.0340	0.0016	0.3232
non-OECD	0.9999^***^	0.0061	−0.0011	−0.0031	−0.2318	0.0019	0.9055

***, ** * *Indicates the significance at 1, 5, and 10%, respectively*.

### Discussion of Findings

#### Economic Growth

Table 10 provides the summary results, it is evident that education, labor and health have no significant impact on economic growth. The insignificance of education is in line with (23–25), who have stated that early education of school has an insignificant impact on economic development. A large number of arguments are given in support of the insignificant relationship between education and economic growth: (i) in the case of underdeveloped and developing countries, the main motive for education is to get high salary positions, instead of possessing high tech skills, (ii) most of the countries follow a traditional educational pattern and they also face a lack of resources, which does not guarantee economic development, however, education in such countries is not a significant contributor in economic growth.

**Table 10:** Summary of results.

	**Economic growth**		**Health**		**Carbon emission**	
	**Long-run**	**Group**	**Long-run**	**Group**	**Long-run**	**Group**
Y	–	–	Insig	Neg	Insig	Pos
E	Insig	Insig	Neg	Neg	Insig	Insig
L	Insig	Insig	Insig	Insig	Insig	Insig
H	Insig	Insig	–	–	Insig	Insig
K	Pos	Pos	Neg	Insig	Insig	Pos
C	Pos	Pos	Pos	Pos	–	–

*Pos represents the positive relationship, Neg shows negative relationship, Insig mentions insignificant relationship*.

The positive dependence of capital and carbon emission on economic development reflects that the alarming situation of carbon emission may be caused by higher dependence on non-renewable energy sources (oil, fossil fuels, and gas) for industrialization and transportation purposes. In the scenario of positive coefficients of capital and carbon emission, it seems that higher capital investment is used for industrialization, which comprises more energy for industrial processing and transportation. However, this higher energy consumption through non-renewable sources and fuel consumption for transportation increases the carbon emission level (119).

#### Health

Short-run and long-run estimations for health issues have reported that economic growth has an insignificant role in controlling health issues, suggesting that higher economic growth does not guarantee an improvement in health conditions and health systems, as many high pace growing economies mostly focus on enhancing business activities, industrialization, urbanization, etc. However, health may have less of a priority, as in such scenario, economic growth, and health issues are insignificant.

The significant and positive coefficient of economic growth is found for high income countries. However, health reforms and education are positively related and considered as investment in human capital. The two of them have their effects on the economic condition of an individual and the state as a whole. Moreover, a person possessing both health and education is more productive and contributing its role in economic development process. The variations in the national incomes of different countries are associated with the health and education of their labors. Moreover, it has also been observed that the countries with low national income may not have enough resources to spend on the health and education sectors. In general words, an individual with low income is not able to consume on education and health. In contrast, investing in the health and education gives a long-term profit as it decreases the levels of poverty and increases the standards of living. Additionally, an individual with better health facilities can invest more time in an education and a job, which increases productivity, and resultantly, there is an increase in economic growth.

Another element that needs to be studied is the effect of education on health. For example; low literacy creates a hurdle in understanding the health policies and the importance of medical treatment (115). Goldman and Smith (116) reported the similar outcomes that the individuals who are educated tend to follow the medical treatments in a more sophisticated manner. Moreover, the individuals who are highly educated and are aware of do's and don'ts spend a considerable amount of time in the health-related activities, such as workouts, and maintain their diets in order to stay healthy (43). In addition to this, a highly educated nation spends a large part of their higher incomes in consuming a healthier standard of living (120). In today's world of technology and pollution, it is necessary for the certain factors to work together to improve health and lifestyle, which are interlinked with educational attainment and technical skills. Patients utilize their prior knowledge to understand their health needs and the instructions, and then to follow them in an effective manner. Moreover, their education helps them to communicate with the health providers in a better way. Health related studies have shown that people with low educational backgrounds were unable to understand the needs of their health.

The results of capital on health are contradicting with the recent studies [such as, (31, 102)], which report that higher capital investments boost the economic progress. Simultaneously, it produces harmful gases, wastes, etc. that are harmful for human health, such as, carbon emission, sulfur dioxide emission, nitrogen dioxide, waste water, etc. that have adverse impact on human health. The results argue that higher capital formation help to increase the economic growth, which increases the urge on the government to provide basic socioeconomic benefits to the general public, such as education and health facilities. However, we can draw a conclusion that higher capital investments lead to increase the economic growth, which reduces the health issues, however, Economic Growth has reported an insignificant coefficient with health issues, so this justification is worthless. Another significant reason for this negative association is education channel; the rise in business activities motivates the rural population to move toward cities, where they can get better education and health facilities that help to reduce the human health problems (116, 120).

Studies have concluded that the carbon emission affects the health of a person adversely (99, 100). Similarly, a large number of studies have also stated a connection between the carbon emission and the health, disease like lungs cancer, asthma and chronic bronchitis, lower respiratory infections, and premature mortality. These diseases are due to direct exposure to the polluted environment (121, 122). It has also been concluded that the countries with a poor condition of environment are likely to face more health issues, such as cardiovascular problems and respiratory problems (123–125). Badamassi et al. (121) also proved that the carbon emissions gave a significant and positive impact on the health issues. It means that the polluted air affects the environment as well as the human health in a negative manner, which lowers the labor productivity.

#### Carbon Emission

This significance of capital to carbon emission is according to our expectation that capital is mainly used for expansion of industrial infrastructure to surge the economic growth. Such industrial expansion also causes higher industrial waste, such as carbon emission. This negative relationship between capital and carbon emission can also be justified through urbanization channel; higher business activities motivate the rural population to migrate toward cities that precipitate the coal and oil consumption in terms of energy generation, transportation, etc., and resultantly, higher coal and oil consumption ejaculate carbon emission.

## Concluding Remarks

The current study is an attempt to examine the nexus among economic growth, education, health and carbon emission, by extending the Solow growth model. For this purpose, we used the data of 161 countries over the period of 2000–2016. In our empirical analysis, we have further categorized the counties according to geographical, income and OECD levels, which provide the cross-group findings for in depth analysis. Empirical estimations have reported the insignificance of education, labor, and health condition on economic growth, while significant and positive coefficients have been reported for capital and carbon emission.

In our full specification empirics, economic growth, and labor have no impact on health condition. Education and capital investment have mentioned significant and negative coefficients, suggesting that higher education and capital investment leads to decreased health issues. On the contrary, carbon emission has significant and positive coefficients, indicating that the environmental degradation process leads to increased health issues. According to carbon emission analysis, economic growth, education, labor, and health have insignificant coefficients. In short-run and group-based analysis, capital investment has presented positive coefficients, signifying that carbon emission is caused by higher capital investments. As a summary of findings, we note that capital formation is the significant contributor of economic growth. Whereas, a significant improvement in education standards helps to mitigate health problems. Similarly, economic growth and capital contribute to increase carbon emissions in regional and group wise analysis. In all of our empirical findings, we find the consistent relationship of variables. More specifically, in GMM estimations, the empirical results indicated no autocorrelation and highlighted that the equation instruments are valid.

To account for the empirical results, we propose policy implications for policy makers to overcome the economic, social, and environmental issues. Capital investment has mentioned a positive relationship with carbon emission and economic growth, indicating that higher capital investment is essential for economic growth, on the other hand, it caused environmental degradation. On this basis, we recommend that industries and government have to promote the energy efficient and green technologies which help to control the carbon emission without effecting the economic development. Specially, East Asia and Pacific and South Asian countries have to motivate the businesses to promote green financing and low-carbon industrial technologies. Simultaneously, there should be heavy carbon tax on industries, as followed by Singapore, China, etc. Second policy implication is related with educational reforms, there is strong need of implementing upgraded, practical and skillful educational curriculum. Such educational reforms, on one side, help to increase the labor productivity, industrial performance, and economic development process. On the other hand, higher educational level enables the public to understand the health and environmental related issues and make them capable enough to overcome these issues.

## Data Availability Statement

The raw data supporting the conclusions of this manuscript will be made available by the authors, without undue reservation, to any qualified researcher.

## Author Contributions

SS: writing of manuscript. MA: collection of data, finalizing the estimations, and review of manuscript. CT: review of manuscript.

### Conflict of Interest

The authors declare that the research was conducted in the absence of any commercial or financial relationships that could be construed as a potential conflict of interest.

## Footnotes

^1^Despite of cross-sectional dependence, we use the first-generation unit root test to robust check the presence of stationarity of variables.
